# Rethinking Barrett's Surveillance When Surveillance Is Most Questioned: Can Dedicated Lists Be Part of the Answer?

**DOI:** 10.1002/ueg2.70286

**Published:** 2026-08-25

**Authors:** Guiomar Moral‐Villarejo, Jacobo Ortiz‐Fernandez‐Sordo

**Affiliations:** ^1^ Nottingham Digestive Diseases Centre and NIHR Nottingham Biomedical Research Centre Nottingham University Hospitals NHS Trust and University of Nottingham Nottingham UK

Oesophageal adenocarcinoma continues to rise across Europe, with Barrett's oesophagus (BO) remaining the only recognised precursor lesion. Endoscopic surveillance has traditionally been the gold standard for detecting dysplasia and early neoplasia; however, its role is now being questioned more than at any point over the past decade. In the last few years, studies from leading groups have demonstrated very limited benefit of surveillance programs. An expert‐centre cohort from Cambridge reported very low disease‐specific mortality under long‐term surveillance [1], while a Dutch community‐based cohort with non‐dysplastic BO found progression risk low enough to support a more conservative, risk‐stratified follow‐up [2].

More recently, we have seen the publication of the first randomised controlled trial evaluating endoscopic surveillance in BO, the Barrett's Oesophagus Surveillance versus Endoscopy at Need Study (BOSS), and even considering all its limitations, it failed to demonstrate an overall survival benefit from two‐yearly surveillance over a 20‐year follow‐up period [3]. Furthermore, everyone with an interest in BO will be already aware of the recently updated Dutch guidelines, which no longer recommend routine endoscopic surveillance for low‐risk patients [4].

For many reviewers and readers, the findings of the BOSS trial are considered to reflect important limitations in how surveillance was delivered during the recruitment period, rather than a failure of the underlying surveillance concept itself [3, 5]. We can then argue than the debate has shifted from whether patients with BO should undergo surveillance to focus on how surveillance should be structured, delivered, and by whom. In this issue, Gamakaranage and colleagues [6] present the largest multicentre comparison published to date of dedicated (DEBO) versus conventional BO surveillance services and are contributing to address some of these questions.

This study analysed more than 2000 procedures performed across six UK hospitals over an eight‐year period. All key performance indicators (KPIs) favoured the dedicated surveillance pathway. The dysplasia detection rate (DDR) was approximately 2.5‐fold higher than in conventional practice (6.9% vs. 2.8%) and 47% more patients had visible lesions documented when scoped by trained endoscopists; more than three times higher than in non‐dedicated lists, this measure may closely reflect lesion detection rate (LDR). Both DDR and LDR represent clinically meaningful outcomes and provide more objective and pragmatic measures than most KPIs. The fundamental rationale of BO surveillance is to identify neoplasia at an early stage, to allow curative minimally invasive endoscopic therapy; detection of dysplasia or visible lesions represents a direct measure of achieving this goal. These findings are consistent with earlier single‐centre and pilot randomised data from some of the same authors [7, 8].

It is worth highlighting one of the findings of this study: the inverse relationship between adherence to the Seattle random‐biopsy protocol and DDR. This most likely reflects endoscopist behaviour: highly skilled lesion‐recognition endoscopists, who comprise most of the dedicated cohort, appear to skip random biopsies for target biopsies when a visible abnormality is identified. This results in lower adherence to the protocol but is paradoxically associated with higher DDR, likely reflecting a higher LDR. If we accept that Barrett's surveillance performed by experts does not require an intensive biopsy protocol, this will support the DEBO approach by reducing procedure time and costs. The value of random biopsies may need to be reconsidered in expert settings where high‐quality inspection is performed. And whether blind biopsies retain meaningful value when no lesions are identified after detailed inspection remains an important question, which the authors rightly highlight for prospective study.

At a time when services are deciding how to respond to the controversial BOSS trial, the increasing evidence questioning surveillance, and the Dutch approach, particularly significant given its leadership and influence in shaping the Barrett's endotherapy landscape as we know it, a coherent picture is emerging: outcomes depend heavily on who performs surveillance, how it is delivered and to which patients. The future of BO surveillance is unlikely to be a binary choice between universal endoscopic follow‐up or abandoning surveillance altogether, but rather a move towards risk‐stratifying patients while centralising care, ensuring that those who undergo endoscopy are managed within Barrett's‐dedicated services by trained endoscopists and in expert centres where endotherapy for early upper GI neoplasia is performed. Non‐endoscopic tests and pan‐oesophageal sampling devices, such as the capsule sponge, are likely to become important tools for refining surveillance pathways in higher‐risk patients [9], while computer‐aided detection tools may play a role in reducing reliance on protocol‐driven blind sampling [10].

Until high‐quality data mature, particularly from the Dutch experience, this study provides a persuasive argument that how BO surveillance is delivered may matter as much as whether it is delivered at all, offering important insights into how future surveillance programmes should be reshaped. Less surveillance overall, but better surveillance where it matters.

## Conflicts of Interest

The authors declare no conflicts of interest.

## Data Availability

Data sharing is not applicable to this article as no datasets were generated or analysed during the current study.
